# The effects of age and other individual factors on radiation induced ESR signals from fingernails

**DOI:** 10.3389/fpubh.2025.1531253

**Published:** 2025-01-15

**Authors:** Samayeh Azariasl, Hiroshi Yasuda

**Affiliations:** ^1^Department of Radiation Biophysics, Research Institute for Radiation Biology and Medicine (RIRBM), Hiroshima University, Hiroshima, Japan; ^2^Phoenix Leader Education Program (Hiroshima Initiative) for Renaissance from Radiation Disaster, Hiroshima University, Hiroshima, Japan; ^3^Graduate School of Biomedical and Health Sciences, Hiroshima University, Hiroshima, Japan

**Keywords:** ESR spectroscopy, fingernail, retrospective dosimetry, radiological emergency response, inter-individual variation

## Abstract

Biodosimetry is crucial for assessing ionizing radiation exposure to guide medical responses. Electron spin resonance (ESR) spectroscopy using fingernails can be effectively used for both occupational and public dose assessments in radiological accidents because of their accessibility and ability to retain stable radiation-induced free radicals. However, despite two decades of research, challenges remain in achieving accurate fingernail dosimetry, mainly owing to the variation in ESR signals among individuals. The purpose of this study was to explore inter-individual differences in ESR signals in fingernails to improve the accuracy and reliability of extremity dosimetry. Fingernail samples were collected from 15 participants (age: 11–64 years), irradiated with X-rays (160 kV, 6.3 mA) at 0, 5, 10, and 20 Gy, and measured using ESR spectroscopy. The effects of individual factors, such as age, sex, health condition, and lifestyle, on radiation-induced ESR signals (RIS) were investigated. Younger participants exhibited stronger RIS intensities and a more linear dose–response relationship. The RIS intensity in female samples tended to be higher than that in male samples. Interestingly, the fingernals of middle-aged donors who regularly took vitamin supplements showed significantly higher ESR signal intensities than those of similar-age donors who did not take supplements. Notable reductions in RIS intensity during storage in a freezer were observed only in older donor samples irradiated at higher doses. These findings underscores the importance of considering age and other individual factors in the calibration for fingernail dosimetry.

## Introduction

Biodosimetry provides crucial information for those unexpectedly exposed to ionizing radiation, aiding in efficient medical responses. The techniques for biodosimetry are categorized into biology-based and physics-based types. The former includes methods that use dicentric chromosome aberrations, cytokinesis block micronuclei (CBMN), fluorescence *in situ* hybridization (FISH), and lymphocyte depletion rate (LDR) (1, 2). The latter measures the radioactivity or radiation-induced free radicals in biological materials collected from a subject that does not rely on biological responses. Electron spin resonance (ESR) or electron paramagnetic resonance (EPR) spectroscopy with tissue samples, such as tooth enamel, bone, and nail, is a common technique for measuring free radicals generated by ionizing radiation in radiological accidents (3–8).

ESR dosimetry using fingernails is a practical approach for promptly providing extremity doses in both occupational exposure (e.g., to X-rays from an irradiation device) and public exposure (e.g., to *γ*-rays from an orphan source) accidents, because of the accessibility, non-invasiveness, and stability of radiation-induced free radicals. The dosimetric characteristics of ESR signals from fingernails following radiation exposure have been investigated for more than two decades, and their effectiveness for retrospective dosimetry has been confirmed under specific conditions (9–18). Fingernail dosimetry is based on the detection of radiation-induced free radicals within the keratin matrix of the fingernails. Keratin is a primary structural protein of the nail, and this fibrous protein is susceptible to ionizing radiation in the formation of radiation-induced free radicals. These sulfur-centered radicals are stable over extended post-irradiation periods, making human fingernails a promising candidate for a natural dosimeter. However, despite extensive investigations in the field of fingernail dosimetry, it remains unclear whether fingernails can be reliably utilized for emergency or retrospective dosimetry mainly because of the difficulty in dose reconstruction attributed to significant individual differences in the radiation sensitivity of the radical formation process.

ESR signals in fingernails can be complex because of the presence of multiple types of radicals, including radiation-induced signals (RIS), mechanically induced signals (MIS), and background signals (BGS). To distinguish these three types of signals and their corresponding components, characteristics such as g-factor, microwave power dependence, and stability, have been examined (19–21). However, the separation of these signals still faces challenges for accurate dose determination, because there are notable inter-individual variabilities in these signals, which can be influenced by factors such as age, health, and lifestyle. Additionally, ESR signals in fingernails can fade over time, and this fading pattern can vary significantly, particularly unless the samples are stored under rigorously controlled conditions (12, 22–26). This feature complicates retrospective dosimetry using fingernals, because the ESR signal intensity measured after a certain period may not accurately reflect the initial radiation exposure. Qantifying these variations across different individuals is crucial for standardizing the dose assessment procedure using fingernal ESR signals in a real accident.

Therefore, in the present study, we attempted to quantify the inter-individual differences in the ESR signals of fingernails in relation to the possible factors underlying these differences. While previous studies have presented the basic principles and procedures of fingernail ESR dosimetry (10, 14, 21, 27), knowledge regarding the extent and sources of variability among individuals is limited. We aimed to fill this gap by systematically analyzing the ESR signals of fingernails collected from physiologically diverse participants, including children, and then by investigating the effects of age and other factors, such as sex, health status, and lifestyles, on the intensity and stability of ESR signals.

## Materials and methods

### Questionnaire survey

A validated questionnaire was distributed to each participant to provide information, such as age, sex, personal custom, dietary habit, health status, medical history, medication and radiation exposure history. Informed consent was obtained from all participants or their parents after they were thoroughly briefed on the study objectives and procedures.

### Sample collection and preparation

Fingernail and toenail samples were voluntarily provided by 15 donors of different ages, including children (11–64 years, Asian type), for this study. Nails were collected during routine hygienic procedures by the donors, pooled, and placed in small sealed plastic bags before being sent to the research laboratory. Prior to the measurement, nail clippings were cut as necessary to fit the size of the ESR sample tube. The dimensions of all the prepared samples were approximately 1–2 mm wide and 4–5 mm long. Without any water treatment, the first ESR measurement (BGS + MIS) was recorded. Then, the nail samples of each donor were soaked in 500 μL of distilled water for 1 h, followed by drying under vacuum (VE-ALL1-8-989-01, AS ONE, Osaka, Japan) for 1 h. This process eliminated any potential pre-experiment, radiation-induced (RIS), or mechanically induced signals (MIS), thereby minimizing the background signal intensity (24, 28). It is worth noting that all nail samples were prepared on the day of the irradiation experiment, and the samples were stored in a freezer (−20°C, 20% humidity) between harvesting and preparation.

### Sample irradiation

**Figure 1:**
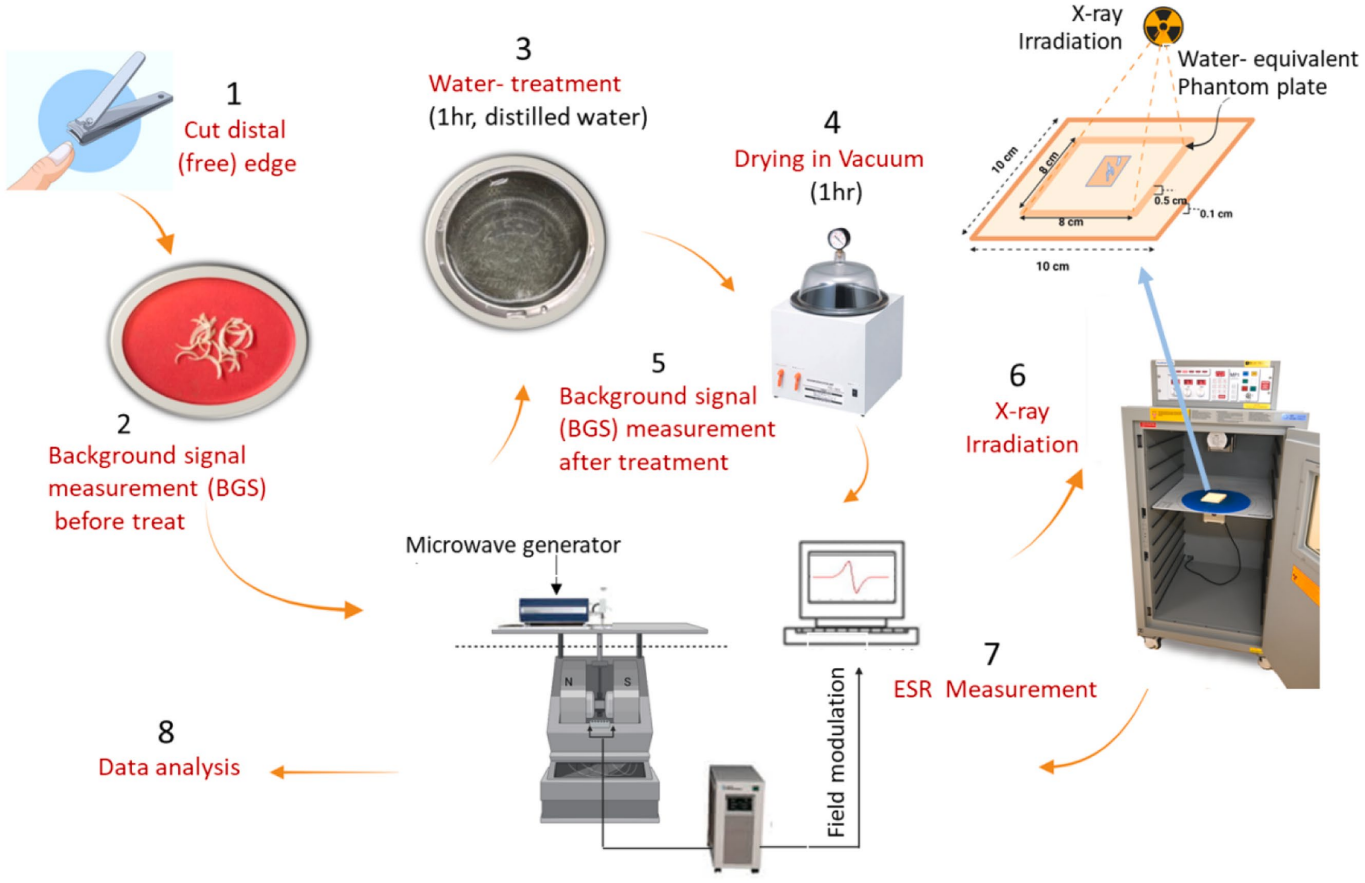
The procedures of preparation of fingernail samples, irradiation with X-rays, and measurements of ESR signals.

For this experiment, we prepared fingernail samples with a total weight of 80 mg (16 pieces for adults and 12 pieces for children) for each donor, and subsequently divided them into four sets (each set including 4–5 pieces with a weight of 18–20 mg). A piece of each donor was irradiated with X-rays (160 kV, 6.3 mA) at 0, 5, 10, or 20 Gy, considering the radiologically concerened dose range. We used toenail samples to confirm the BGS intensities of selected children’s samples because of the insufficient amount of fingernail samples, as no differences were observed between the properties of fingernail and toenail samples in a previous study (24). A commercial X-ray irradiator (Cabinet X-ray System Model 43855F with CP160 Option, Faxitron X-ray LLC, Illinois, United States) was used to irradiate samples with X-rays at a dose rate of 2.7 Gy min^−1^ (Figure 1).Therefore, three sets of samples for each donor were irradiated to investigate the dose–response, and one set of samples from each individual was kept unirradiated as a control and measured at the same time as the irradiated sets.

### ESR measurement

The ESR spectra were measured with a X-band ESR spectrometer (JES-FA100, JEOL Inc., Chiba, Japan) equipped with an ES-UCX2 cavity resonator. Each sample was measured using 5 mm diameter thin-walled quartz ESR tubes. All measurements were performed under ambient conditions with 30% ± 5% relative humidity at 20–24°C at room temperature with a central magnetic field of 336 mT. Parameters for spectra data acquisition were as follows: microwave frequency 9.4 GHz; microwave power 1 mW; sweep width 10 mT; sweep time 30 s; modulation width 0.4 mT; time constant 0.03 s; total number of scans 10; and total recording time 10 min (which included both sample loading and spectrometer tuning times). The Mn^2+^:MgO internal standard, positioned at the base of the cavity, served as a spectral reference for calibration during measurement. Quantitative analysis of the spectra, including baseline corrections and the measurement of peak-to-peak ESR signal amplitudes, was conducted using the A-System Data Processing software (version 3.9.2.0, JEOL Resonance Inc., Chiba, Japan). The ESR measurements were repeated three times for each sample while being rotated in the tube. Measurements were conducted at six post-irradiation times: 0.05, 1, 2, 3, 5, and 7 days. To prevent rapid signal fading, the samples were consistently stored in a freezer at −20°C with humidity levels maintained around 20% between successive measurements immediately after irradiation. A humidity logger (LR5001, Hioki, Nagano, Japan) equipped with a built-in sensor (LR9504, Hioki, Nagano, Japan) and a communication adapter (LR5091, Hioki, Nagano, Japan) was used to monitor the humidity inside the freezer.

## Results

### ESR spectra

**Figure 2:**
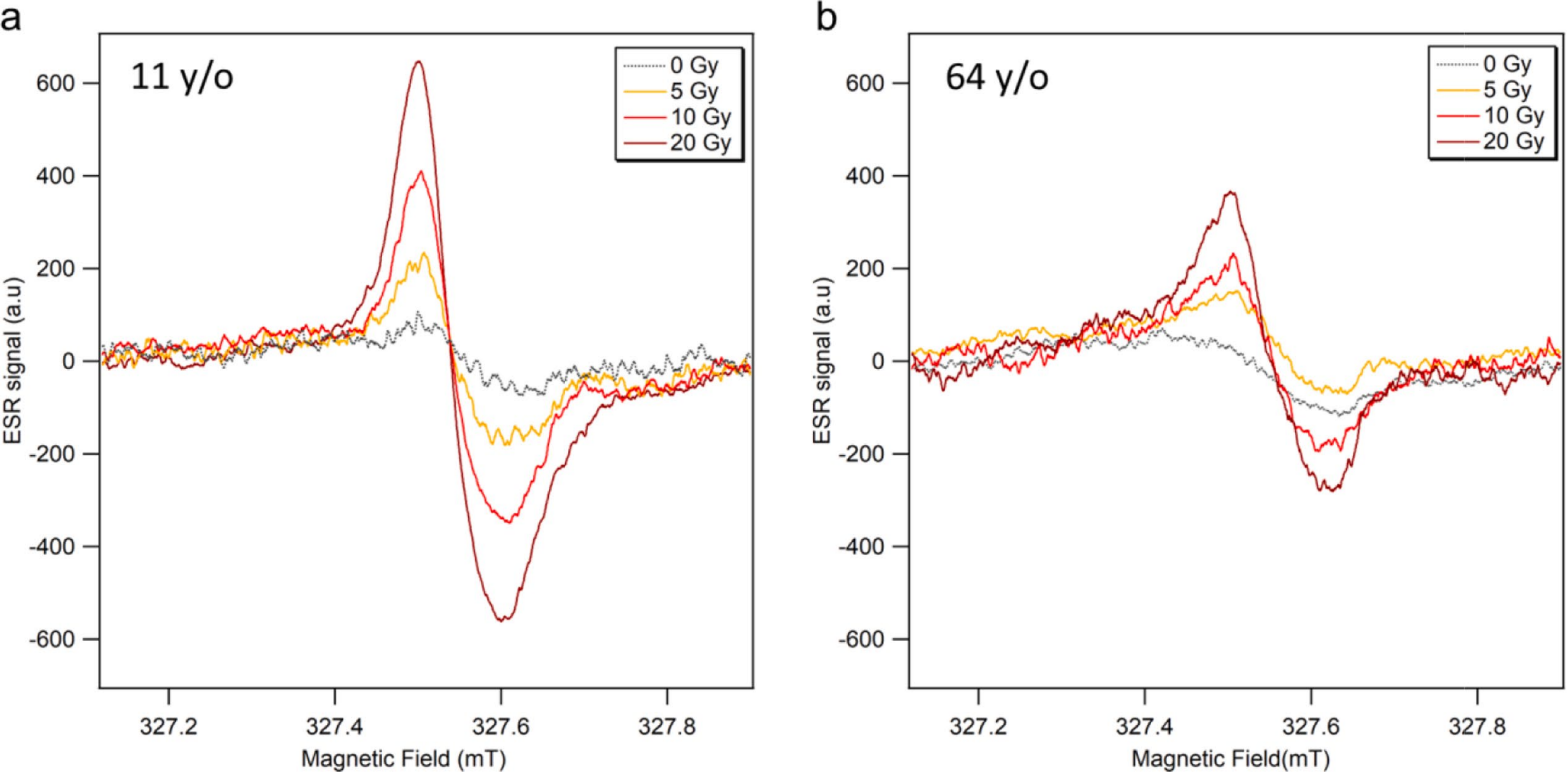
ESR signal spectra of fingernails collected from **(A)** 11 years old and **(B)** 64 years old female donors, measured soon after water treatment (0 Gy) and subsequent irradiations with X-rays (160 kV, 6.3 mA) at 5, 10 and 20 Gy.

The ESR spectra of fingernails collected from the youngest (11 years old, female) and oldest (64 years old, female) donors are shown in Figure 2. These data were obtained immediately after water treatment (0 Gy), followed by irradiation with X-rays (160 kV, 6.3 mA) at three radiation doses (5, 10, and 20 Gy). Each data point represents the average of three ESR spectra measurements. As seen in these figures, the RIS intensities of the fingernails of children were significantly higher than those of the older adult donors’ samples.

### Dose responses

**Figure 3:**
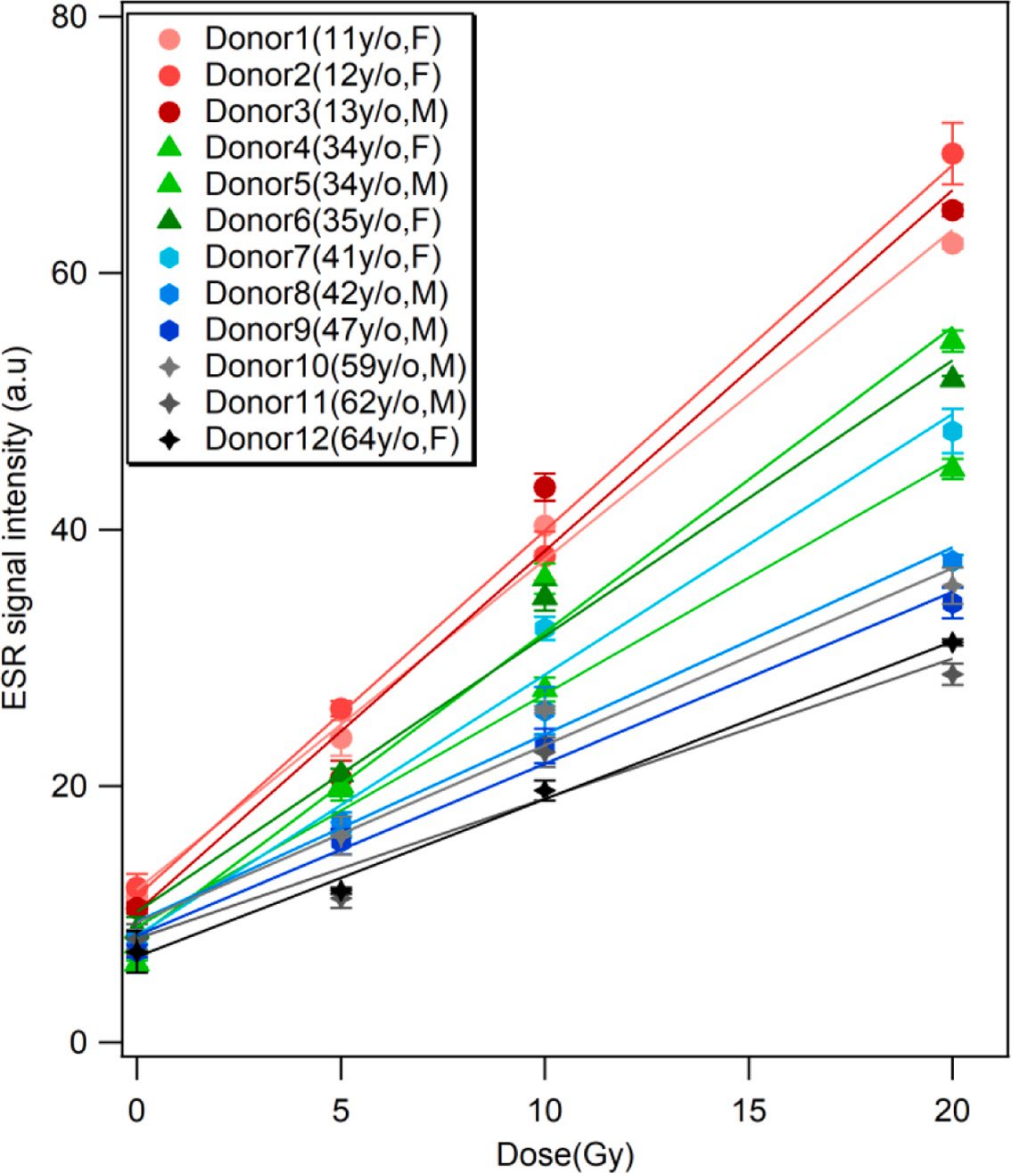
Dose responses of ESR signal intensity of fingernails collected from 12 healthy donors with different ages. The samples were irradiated with 160 kV X-rays at 5, 10, and 20 Gy. The background signals were not subtracted. Each age group is represented by different markers, and the marker color becomes darker with the increasing age in the same group. Each data set is fitted with a linear function.

The dose responses of the ESR signal intensities from the fingernails of the 12 donors are shown with linear fitting curves in Figure 3. The data from three donors were excluded here because they regularly took vitamin supplements; these data are presented in the section of ‘Effects of vitamin supplements. Each plot is the average of three measurements, which was performed immediately after water treatment (0 Gy) and 1 h after subsequent X-ray irradiations (5, 10 and 20 Gy), with the error bar indicating one standard deviation. The signal intensity presented in this study was depicted as the peak-to-peak amplitude of the major signal singlet, as adapted by Sholom et al. (23). All ESR intensities (peak-to-peak amplitudes) were normalized to the unit weight of the sample throughout this study. The data points were well fitted with a linear function *y = mx + y_0_*, where *m* is the slope and *y_0_* is the intercept. The values of the linear regression parameters and determination coefficient (*R^2^*) calculated for the ESR signal intensities of each donor’s fingernails are summarized in Table 1.

**Table 1:** Linear fitting parameter and *R*^2^ values for the ESR signal intensities of each donnor fingernails.

Donor number	Age (y/o)	Sex	Linear fitting equation	*R*^2^ value
Donor 1	11	F	*y* = 2.5x +11.913	0.99
Donor 2	12	F	*y* = 2.85x + 11.382	0.99
Donor 3	13	M	*y* = 2.81x + 10.175	0.97
Donor 4	34	F	*y* = 1.816x + 8.9739	0.99
Donor 5	34	M	*y* = 2.380x + 8.1743	0.97
Donor 6	35	F	*y* = 2.151x + 10.165	0.98
Donor 7	41	F	*y* = 2.033x + 8.3149	0.97
Donor 8	42	M	*y* = 1.407x + 9.6129	0.98
Donor 9	47	M	*y* = 1.341x + 8.304	0.98
Donor 10	59	M	*y* = 1.382x + 9.3305	0.97
Donor 11	62	M	*y* = 1.091x + 8.0979	0.92
Donor 12	64	F	*y* = 1.23x + 6.6663	0.99

Although linear relationships were confirmed in all cases, as the *R*^2^ values were significant (ranging from 0.92 to 0.99), the children fingernails (Donors 1–3) showed greater linearities in dose response over the entire dose range (0–20 Gy). Another interesting observation from the dose–response data (Figure 3; Table 1) was that the linearity of the dose–response curves tended to decrease with increasing age. As results, the ESR signal intensities of younger donors’ fingernails were clearly higher than those of older donor samples at higher doses.

### Effects of age and sex

**Figure 4:**
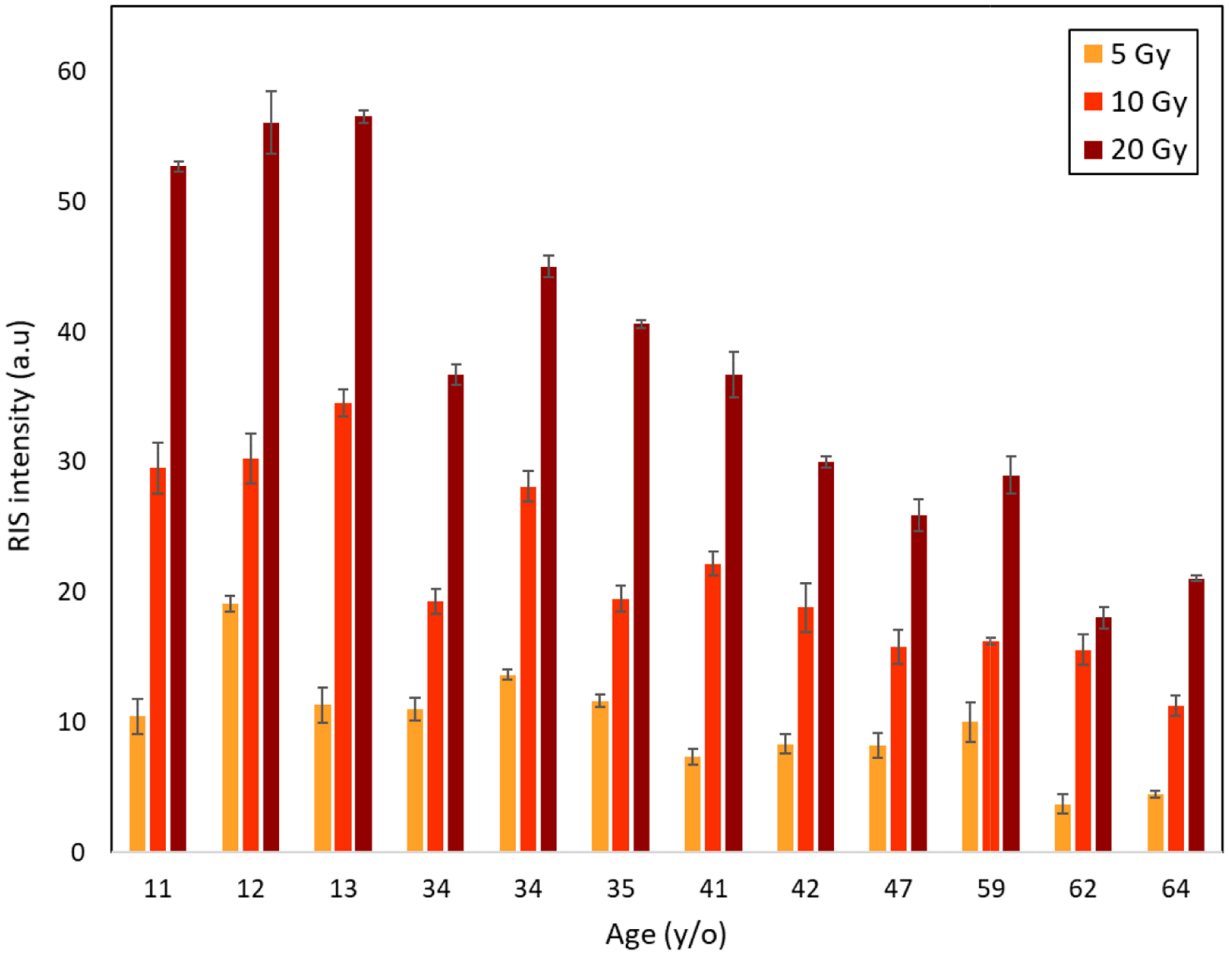
Age dependence of RIS intensities of fingernails collected from 12 donors aged from 11 to 64 years old after irradiations with X-rays at 5, 10 and 20 Gy.

Figure 4 shows the RIS intensities (i.e., post-irradiation increments in the ESR signal intensity) of the fingernails 1 h after irradiation with X-rays at 5, 10, and 20 Gy. Error bars indicate the standard deviations of three measurements. RIS intensity tended to gradually decrease with increasing age at the same dose level. Another interesting observation was that the reduction pattern of RIS intensity was nearly the same despite the dose difference. For instance, 79.8, 89.7 and 85.8% reductions in RIS intensity were observed between the youngest (age: 11 years) and oldest donors (64 years) at 5, 10, and 20 Gy, respectively. Although there was some variability in these patterns, the results obtained in this study indicated that the RIS intensity decreased with increasing age as an overall trend.

**Figure 5:**
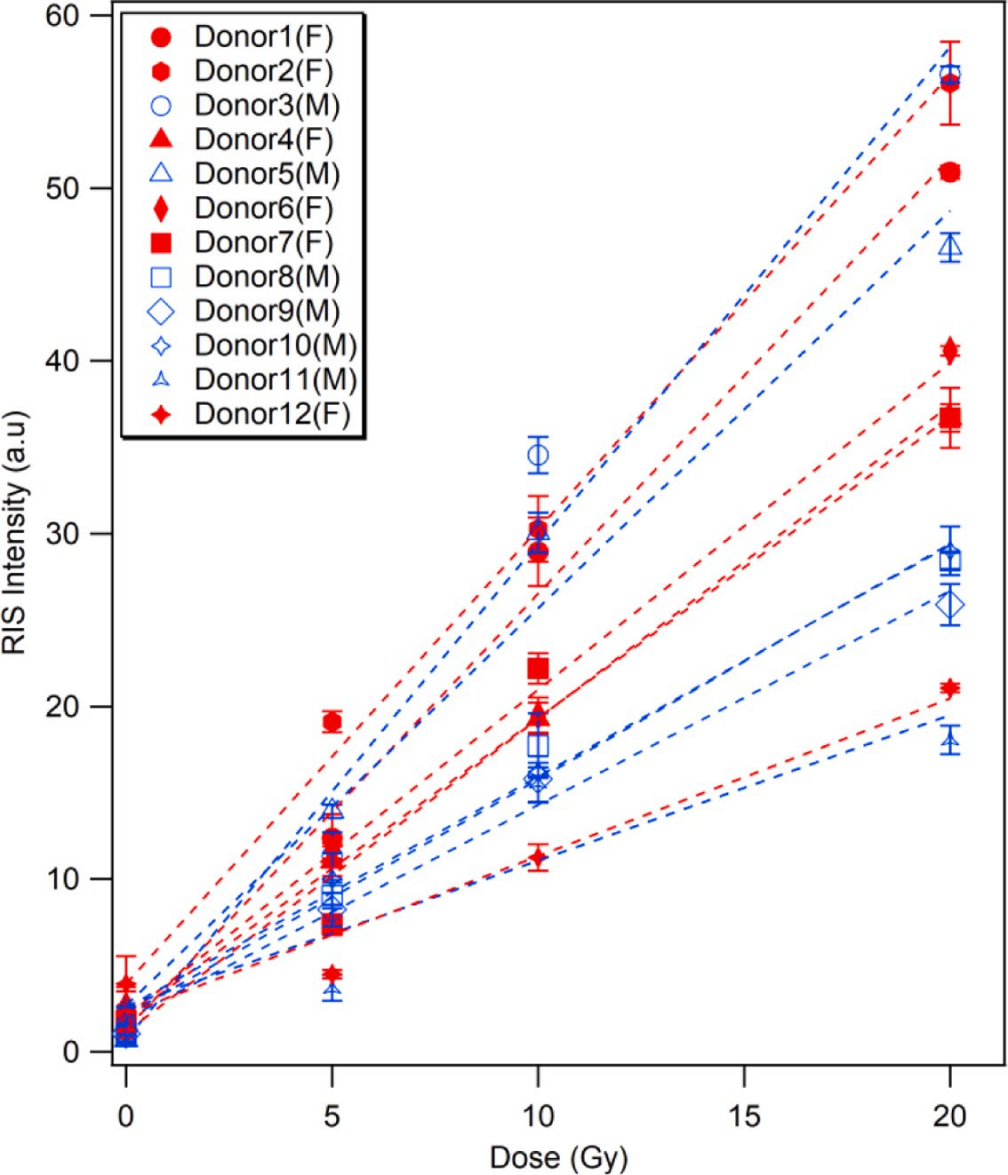
Sex dependence of radiation-induced ESR signal.The RIS intensities of fingernails collected from 12 donors prior to and after irradiation with X-rays (5, 10, and 20 Gy). Plots are colored according to sexs of the donors; red markers/lines indicate female donors, while blue markers/lines male donors.

The next comparison, shown in Figure 5, is related to the sex dependence of RIS intensity among the 12 donors (6 males and 6 females). Overall, female fingernails showed higher RIS intensities than male fingernails, particularly in middle-aged donors at higher doses, although these differences were not statistically significant. However, given the limited magnitude of these differences, further studies are required to fully understand the potential sex-related variations in fingernail radiosensitivity, particularly at lower doses.

### Effects of vitamin supplements

**Figure 6:**
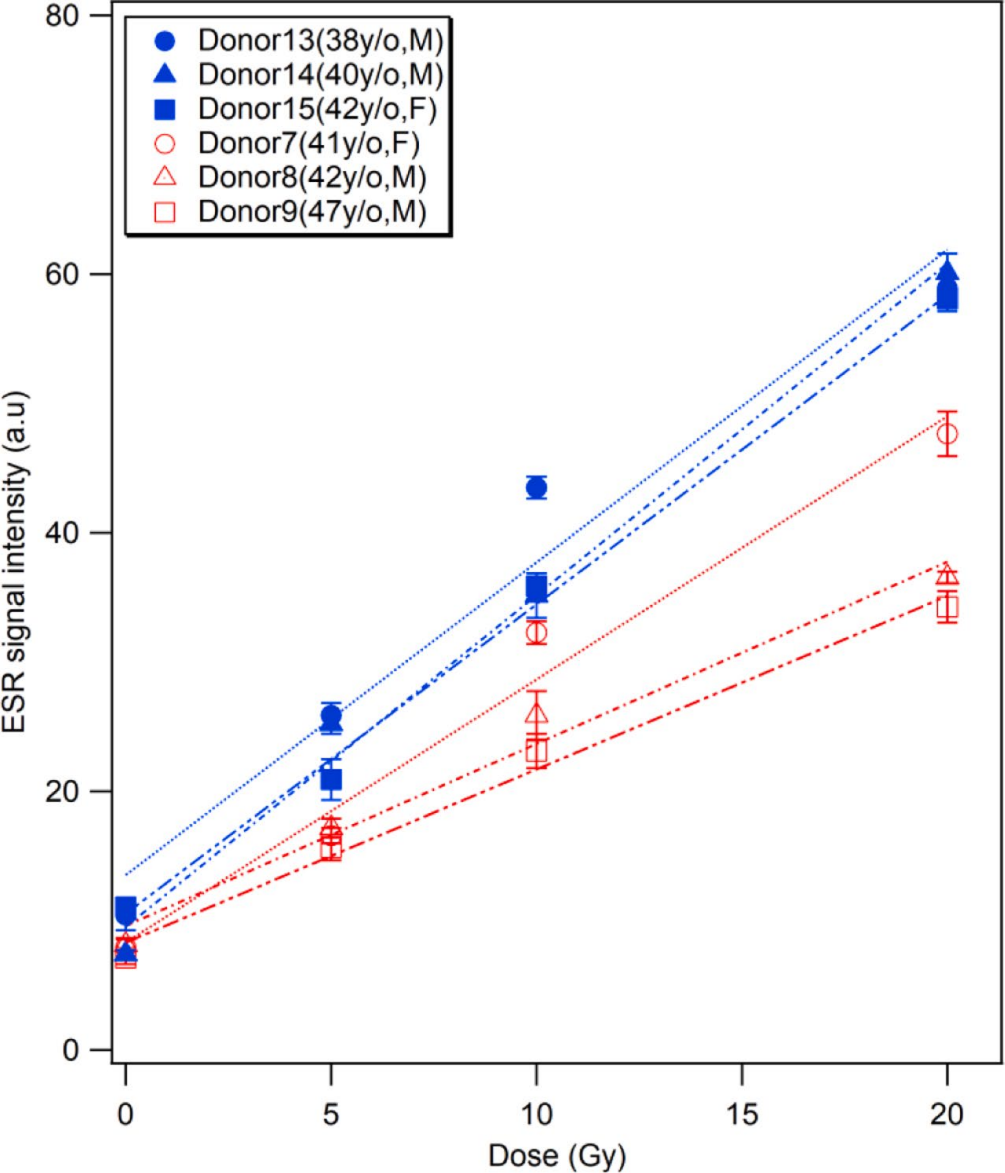
Effects of vitamin supplements. Comparisons of the ESR signal intensities of the fingernails collected from three middle-aged donors who regularly took multivitamin supplements (Donors 13–15, in blue) and those with similar ages who do not take any supplements (Donors 7–9, in red).

Analyzing the answers from the donors to the questionnaire about their lifestyles, diet, and medication on a daily basis, we found that three middle-aged donors (age:41–47 years) showed higher BGS and RIS intensities at different doses than other donors of similar ages (38–42 years), as shown in Figure 6. Although the individuals of middle age group showed considerable variation (9–26% on average), the difference in RIS intensity between these donors and others (43–61% on average) was more significant. Accordingly, we checked the health-related information of these donors and compared them with other donors, and unexpectedly found that all three donors orally received multivitamin supplementation on a daily basis for at least 3 months. Considering this fact, we excluded these donors from the dose response analysis in this study (Figures 3–5). While further investigations should be conducted using more samples from different donors who take vitamin supplements, we expect that this interesting finding would raise the possibility of achieving an individual calibration for fingernail ESR dosimetry based on the quetionnaire survey.

### Signal stability during the experiment

**Figure 7:**
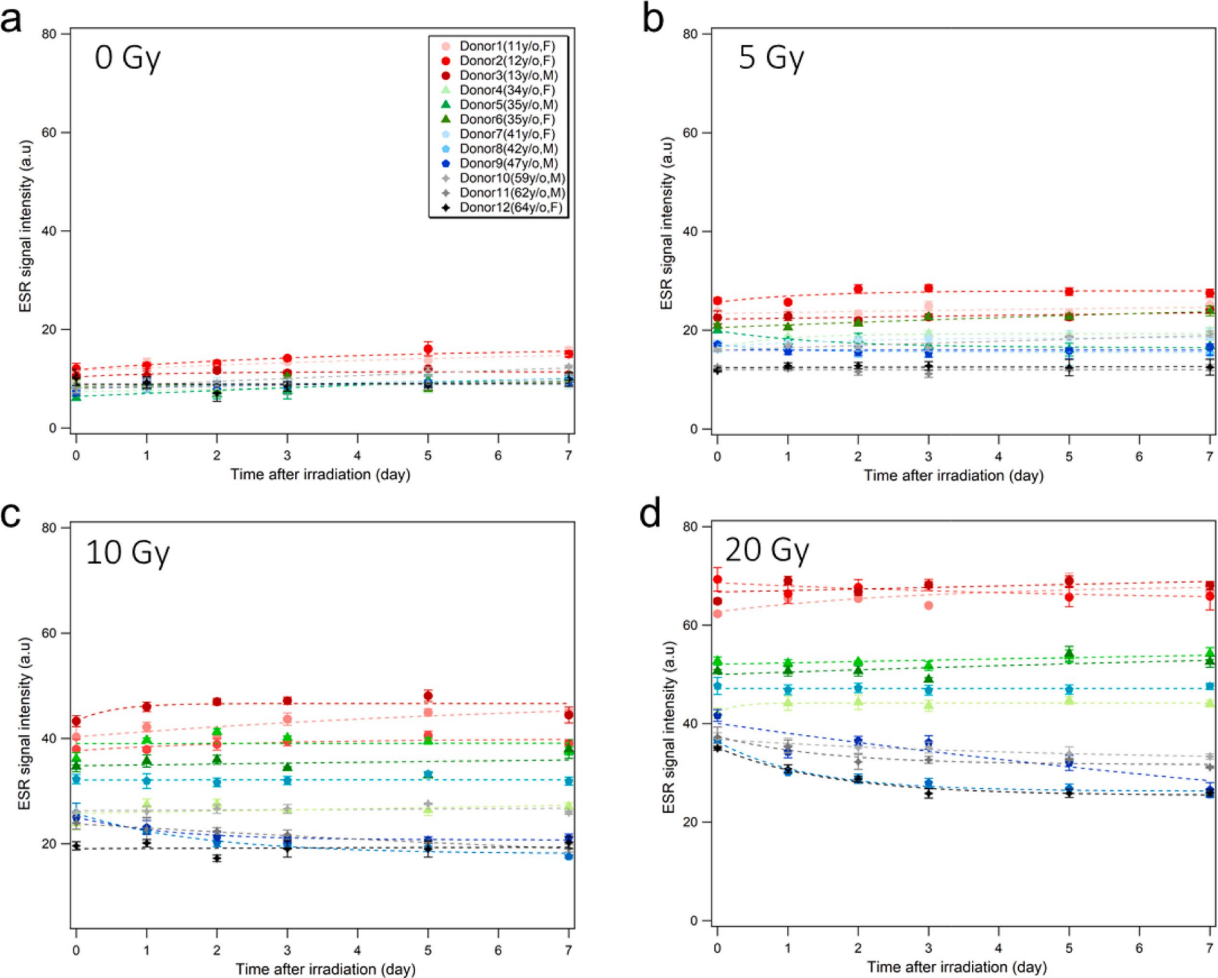
Stability of ESR signal intensity during storage.Time changes of the ESR signal intensities of the fingernails collected from 12 healthy donors without irradiation **(A)** and after X-ray irradiation at 5 Gy **(B)**, 10 Gy **(C)**, and 20 Gy **(D)** during storage in a freezer (−20°C, 20% humidity) over 1 week. The dash lines are empirically fitted regression curves.

Figure 7 shows the results of time changes of ESR signals from fingernails of 12 donors during 7-days storage in a freezer (−20°C, 20% humidity) after X-ray irradiations at four different doses (0, 5, 10, and 20 Gy). Overall, fingernail ESR signals showed good stability in the freezer for over 1 week, as observed in a previous study (26). It should be noted that a slight decay in RIS was observed 24 h after irradiation in aged donors, especially at higher doses (10 and 20 Gy), while younger donor samples showed evident stabilities at all dose levels.

## Discussion

The individual responses of ESR signals in human fingernails of different ages exposed to different doses up to 20 Gy were investigated. The dose–response curves of the ESR signals showed significant variation among the donors (Figures 3, 4). The results indicated that RIS intensity could be affected by donor’s age with a tendency of decreasing with increasing age. The reason for these observations is unclear at present, while we observed that the nail samples from the aged donors were thicker, more brittle, and had a yellowish color compared to the other samples. This variation might be attributed to age-related changes in fingernail components, which are potentially influenced by environmental and chemical factors such as sunlight, daily washing, and the use of nail polish. In addition, biomolecular variations in human fingernails may be attributable to variations related not only to age, but also to other factors such as sex, lifestyles, or physiological differences in the individuals who donated fingernail samples. However, significant variability was found between the dose–response curves among donors of a similar age, and this variation was more evident in the middle-aged group. This implies that the formation of radiation-induced free radicals is not only dependent on age but can also be affected by other factors such as sex, lifestyle, diet, and physiological conditions.

Our finding suggests a moderate difference in the radiosensitivity of fingernails between male and female donors, with female participants exhibiting a higher peak-to-peak amplitude of the RIS, especially in middle-aged donors (Figure 5). These differences were not statistically significant, particularly at lower irradiation doses, indicating that sex might not play a dominant role in determining the radiosensitivity under these conditions. Interestingly, the trend toward higher RIS intensity in females was more pronounced with increasing doses, suggesting that sex-related factors might exert a more substantial influence at higher doses. The absence of significant differences at lower doses emphasizes the need for larger sample sizes and more controlled studies to elucidate the underlying mechanisms of these potential sex-dependent effects. As we only used a limited number of subjects (12 donors) and it is known that different factors could affect the ESR signals of fingernails (23, 27, 29), we plan to conduct further investigations using more fingernail samples collected from a larger number of donors with different individual factors, such as age, lifestyle, dietary habit, and physiological health condition, to validate the universality of the findings obtained in this study.

Related to universality, there is a question regarding the effects of the radiation quality. In this study, we used X-rays mainly because hand-exposure accidents with high-dose X-rays are common among surgeons (30). Prior studies suggest that fingernails exhibit comparable sensitivity to both X-rays and *γ*-rays; for example, the dose responses of the RIS intensities for X-rays and γ-rays showed similar, simply increasing curves (29), which implies that both photon radiations are expected to produce chemically similar radicals in keratin structures (31). Nevertheless, variations in the radical yield and ESR signal intensity may occur because of variable interactions through different ionization processes. Further investigations are needed to clarify whether the dose responses curves obtained using X-rays can be directly applied to γ-rays or other radiation sources.

Unexpectedly, we found that daily vitamin supplementation by donors could affect the RIS intensity (Figure 6). This is the first finding in the research on fingernail dosimetry over approximately two decades, despite some studies that showed the long-term effects of mineral vitamin composites, including vitamins B1-B6, A, D3, calcium, iron, zinc, etc. to strengthen the nail structure (32, 33). Thus, it can be hypothesized that vitamins could increase the bonds in *α*-keratin molecules trap free radicals in fingernails, which would be affected by individual factors (age, sex, lifestyle, diet, etc.). An important consideration in fingernail ESR dosimetry is the potential influence of high antioxidant intake on the dose estimation. Our results showed that donors with regular multivitamin supplementation, which often includes antioxidants, exhibited a steeper dose–response curve. This suggests that antioxidants may alter radical dynamics, potentially affecting RIS intensity.

Given these findings, using the additive dose method in such cases could lead to underestimation of the absorbed dose. This underscores the need for careful calibration and consideration of individual factors, such as antioxidant intake, to ensure accurate dose assessments. Further studies are needed to explore how antioxidants influence radical formation and to improve the accuracy of fingernail dosimetry in diverse populations.

The time stability of RIS intensity is another important factor in fingernail dosimetry because the signals depend strongly on the storage conditions in terms of temperature and humidity. Several studies have explored the stability of ESR signal intensity under different storage conditions, and observed that freezing nail samples at a low (sub-zero) temperature was effective in maintaining the stability of RIS and BGS intensities (12, 22–24, 26, 28). The results of the present study support these previous findings. In addition, we found that the ESR signal intensities of older donor fingernails showed notable declines for a few days post-irradiation even in a freezer, particularly with high-dose irradiation (≥10 Gy); such signal declines were not observed in younger donors. These findings imply that the fingernails of older people have weaker powers to retain the free radicals generated by radiation. Moreover, according to the Figure 7, there was a slight increase in the number of radicals in young donors during storage in the freezer. Actually, we observed in a previous study that younger donor’s post-irradiation samples stored in freezer and refrigerator showed slight increase in first days and became stable until 1 month in these conditions (26). These results are attributable to possible mechanical changes in the spongy structure of fingernail tissue at lower temperature; freezing slowly allows the water molecules in the tissue to line up during the transition and form crystal, which could affect mechanically induced signals (MISs). According to Reyes et al. (12, 19), two signals originate from the mechanical stress on the fingernail cut and are consequently referred to as the first mechanically-induced signal (MIS1) and second mechanically induced signal (MIS2). The origin of MIS1 is thought to be linked to the formation of electron dipoles at S-S bridges following elastic deformation. On the other hand, MIS2 is believed to stem from the plastic deformation of the keratin helix, where electron dipoles or holes develop in the cystine backbone chains during the cutting process, indicating that MIS1 fades over time, whereas MIS2 showed a slight increase until it reaches a relatively stable state. Therefore, a similar increase we observed in the number of radicals in the unirradiated sample can be attribulted to the MIS2. However, it is currently difficult to provide conclusive statements owing to the unstable behaviors of free radicals affected in complicated manners by the nail structure, sulfur bonds, age, thickness, surface area, mechanical stress, and water content.

## Conclusion

In conclusion, the results obtained from the analyses of fingernails collected from 15 donors indicated that the radiation sensitivity of fingernail ESR signal intensity changed with age and other individual factors. The dose response of radiation-induced signal (RIS) intensitiy showed a general tendency to slope more gently in older donors, which implies that the age of the donor is negatively related to the ability to retain free radicals generated by ionizing radiation in fingernails. This implication was supported by the one-week observation of ESR signal intensities of fingernails stored in a freezer, in which only older donor samples irradiated at higher doses (≥10 Gy) showed notable decreases in RIS intensity. Regarding other individual factors, we first found that the intake of vitamin supplements significantly increased ESR signal intensities in middle-aged donors. More comprehensive studies to quantify the effects of sex, diet, lifestyle, and physiological conditions are required for practical applications of the fingernail ESR dosimetry technique in real situations.

## Data Availability

The raw data supporting the conclusions of this article will be made available by the authors, without undue reservation.
